# Neural responses to threat and reward interact to predict stress-related problem drinking: A novel protective role of the amygdala

**DOI:** 10.1186/2045-5380-2-19

**Published:** 2012-11-14

**Authors:** Yuliya S Nikolova, Ahmad R Hariri

**Affiliations:** 1 Laboratory of NeuroGenetics, Department of Psychology & Neuroscience and Institute for Genome Sciences & Policy, Duke University, NC 27708, Durham, USA

**Keywords:** Amygdala, Ventral striatum, Threat, Reward, Stress, Alcohol

## Abstract

**Background:**

Research into neural mechanisms of drug abuse risk has focused on the role of dysfunction in neural circuits for reward. In contrast, few studies have examined the role of dysfunction in neural circuits of threat in mediating drug abuse risk. Although typically regarded as a risk factor for mood and anxiety disorders, threat-related amygdala reactivity may serve as a protective factor against substance use disorders, particularly in individuals with exaggerated responsiveness to reward.

**Findings:**

We used well-established neuroimaging paradigms to probe threat-related amygdala and reward-related ventral striatum reactivity in a sample of 200 young adult students from the ongoing Duke Neurogenetics Study. Recent life stress and problem drinking were assessed using self-report. We found a significant three-way interaction between threat-related amygdala reactivity, reward-related ventral striatum reactivity, and recent stress, wherein individuals with higher reward-related ventral striatum reactivity exhibit higher levels of problem drinking in the context of stress, but only if they also have lower threat-related amygdala reactivity. This three-way interaction predicted both contemporaneous problem drinking and problem drinking reported three-months later in a subset of participants.

**Conclusions:**

These findings suggest complex interactions between stress and neural responsiveness to both threat and reward mediate problem drinking. Furthermore, they highlight a novel protective role for threat-related amygdala reactivity against drug use in individuals with high neural reactivity to reward.

## Findings

Increased amygdala reactivity to threat has been consistently associated with heightened risk for mood and anxiety disorders [1]. In contrast to this heightened risk, a few studies have suggested that threat-related amygdala reactivity may buffer risk for drug abuse. Specifically, one study reported that individuals at high familial risk for alcoholism exhibit relatively reduced threat-related amygdala reactivity [2]. The authors speculate that this pattern may indicate reduced sensitivity to the harmful consequences of excessive alcohol use in those at risk.

Consistent with these findings, a recent study has linked a genetic variant conferring increased risk for drug abuse [3] with relatively decreased threat-related amygdala reactivity [4]. Interestingly, the same genetic risk variant was associated with heightened reward-related reactivity of the ventral striatum (VS), a neural phenotype associated with both risk for and pathophysiology of drug abuse [5,6]. These data suggest a potentially synergistic effect of threat-related amygdala reactivity and reward-related VS reactivity in precipitating drug abuse risk. In addition to variability in these neural phenotypes, drug abuse risk is moderated by environmental factors, such as recent life stress [7]. Both VS and amygdala function are also affected by stress [8], suggesting that complex interactions between these neural circuits may contribute to variability in stress-related risk for drug abuse.

**Figure 1:**
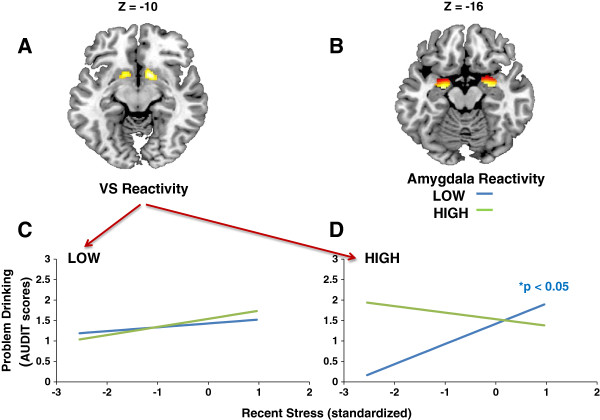
**Amygdala and VS reactivity interact with recent stress to predict problem drinking.** (**A**) Statistical parametric map illustrating mean bilateral threat-related amygdala reactivity (left: x=−22, y=−6, z=−18, *t*=19.76, *p*<0.000001, k_E_=173; right: x=28, y=−4, z=−20, *t*=20.16, *p*<0.000001, k_E_=199). (**B**) Statistical parametric map illustrating mean bilateral reward-related VS reactivity (left: x=−12, y=10, z=−10, *t*=6.19, *p*=3.07 x 10^-7^, k_E_=357; right: x=12, y=10, z=−8, *t*=7.31, *p*=1.03 x 10^-9^, k_E_=383). Activation clusters in (A) and (B) are overlaid onto canonical structural brain images in the axial plane. (**C**) Among participants with low VS reactivity, (1 SD below the mean), recent stress (LESS Highest Impact) was not associated with increased problem drinking (total scores on the AUDIT; square root transformed) regardless of amygdala reactivity. (**D**) For participants with high (1 SD above the mean) VS reactivity, recent stress predicted significant increases in problem drinking only for those who also had relatively low (1 SD below the mean) amygdala reactivity (blue line). Plotted values are adjusted for sex, age and race/ethnicity.

Here, we explore the interactions of recent life stress, threat-related amygdala and reward-related VS reactivity in predicting variability in self-reported problem drinking in a sample of 200 young adults. We focused on drinking because alcohol is the most commonly used and abused drug in adolescents and young adults [9], and its use is often triggered by stress [7]. Using two well-characterized BOLD fMRI paradigms ([10] Figure 1A-B), we quantified threat-related amygdala and reward-related VS reactivity. Recent life stress and problem drinking were assessed using the Life Events Scale for Students (LESS, [11]) and the Alcohol Use Disorder Identification Test (AUDIT, [12]), respectively. Based on prior research, we predicted that higher threat-related amygdala reactivity would protect against increased problem drinking in the context of stress, particularly in those whose risk is exaggerated by higher reward-related VS reactivity.

As expected [7], there was a significant positive correlation between recent stress and problem drinking (r=0.22, p=0.004). Critically, however, this relationship was moderated by amygdala and VS reactivity (Figure 1A-B). Specifically, a three-way interaction predicting problem drinking emerged between recent stress, left amygdala reactivity, and left VS reactivity (ΔR^2^=0.035, b=−0.26, p=0.012). Among participants with low VS reactivity (1 SD below mean; Figure 1C), stress did not predict any increases in drinking, regardless of amygdala reactivity. Among participants with high VS reactivity (1 SD above mean), who are likely to be at increased risk for drug abuse [5], stress predicted increased problem drinking only for those who also had low amygdala reactivity (1 SD below mean; Figure 1D). This three-way interaction remained significant after controlling for gender, age, and race/ethnicity (ΔR^2^=0.031, b=−0.25, p=0.012). There was no such interaction for right VS or amygdala reactivity (p values > 0.10), and no significant main effects of either amygdala or VS reactivity on problem drinking (p values > 0.14).

Demonstrating the specificity of these findings to recent, as opposed to early, life stress, the three-way interaction remained significant when total scores from the Childhood Trauma Questionnaire [13] were added as an additional covariate (left VS: R^2^=0.033, b=−0.25, p=0.011). Furthermore, childhood trauma did not interact with amygdala or VS reactivity to predict problem drinking (p values > 0.63). Finally, the same three-way interaction emerged in a subsample of participants (N=85) who completed a three-month follow-up assessment of stress and problem drinking (without covariates: ΔR^2^=0.085, b=−0.365, p=0.008; with covariates: ΔR^2^=0.063, b=−0.324, p=0.019). The temporal stability of this interaction suggests that stress-related problem drinking reflects rather than affects the relative neural responsiveness to threat and reward.

An important caveat to consider when interpreting these findings is the possibility that participants drinking more alcohol may experience more stressful life events partially as a result of their increased drinking, rather than the other way around. Since our measures of stress and problem drinking are based on retrospective self-report spanning the past 12 months, the directionality of the association between stress and drinking cannot be determined on the basis of these analyses. Thus we cannot rule out the alternative interpretation that individuals with high VS reactivity and low amygdala reactivity are more likely to experience highly impactful stressful life events in the context of problem drinking. This interpretation would be consistent with a heightened drive to pursue immediate rewards, coupled with a reduced ability to recognize and avoid threat in those individuals.

Limitations notwithstanding, we provide novel evidence that recent life stress is associated with increased problem drinking only in individuals with higher reward-related VS reactivity and lower threat-related amygdala reactivity. Consistent with the relative temporal stability of amygdala [14] and VS [15] reactivity, the interactions between these neural phenotypes and recent life stress predicted future problem drinking in a subset of participants. This finding suggests that the pattern we observe spans longer periods of time and may be useful in identifying individuals at particularly high risk for developing alcohol and possibly other substance use disorders in the wake of stress. Future research identifying factors that predict the observed variability in neural responsiveness to threat and reward (e.g., functional genetic polymorphisms) can inform the development of biomarkers for drug abuse risk and interventions targeting these specific intermediate phenotypes.

## Abbreviations

AUDIT: Alcohol Use Disorder Identification Test; BOLD fMRI: Blood oxygen level dependent functional magnetic resonance imaging; LESS: Life Events Scale for Students; VS: Ventral striatum.

## Competing interests

YSN and ARH declare no competing interests.

## Authors’ contributions

YSN and ARH designed this study. YSN conducted the statistical analyses and drafted the manuscript. ARH edited the manuscript. Both authors read and approved the final manuscript.
